# Rb_2_Ca_2_Si_2_O_7_: a new alkali alkaline-earth silicate based on [Si_2_O_7_]^6−^ anions

**DOI:** 10.1107/S2053229625001196

**Published:** 2025-02-17

**Authors:** Volker Kahlenberg

**Affiliations:** a Institute of Mineralogy and Petrography, University of Innsbruck, Innrain 52, Innsbruck, Tyrol, A-6020, Austria; University of the Witwatersrand, South Africa

**Keywords:** crystal structure, Rb_2_Ca_2_Si_2_O_7_, pyrosilicate, sorosilicate, rubidium, calcium

## Abstract

Synthesis experiments were conducted in the ternary Rb_2_O–CaO–SiO_2_ system, resulting in the formation of a hitherto unknown com­pound with the com­position Rb_2_Ca_2_Si_2_O_7_, *i.e.* dirubidium dicalcium pyrosilicate. Single crystals of sufficient size and quality were recovered from a starting mixture with an Rb_2_O:CaO:SiO_2_ molar ratio of 2:1:3. The educts were confined in a lid-covered platinum crucible and gradually cooled from 1050 °C at a rate of 0.3 °C min^−1^ to 800 °C before being finally quenched in air to ambient conditions. The crystal structure was investigated at −80 and 15 °C from single-crystal X-ray diffraction data, with structure determination performed using direct methods. The com­pound was found to be of ortho­rhom­bic symmetry, belonging to the space group *Pmmn* (No. 59), with *a* = 5.7363 (6), *b* = 13.8532 (12), *c* = 9.9330 (10) Å, *V* = 789.34 (13) Å^3^ and *Z* = 4 (at 15 °C). The final refinement calculations at ambient tem­per­a­ture converged at *R*1 = 0.030 and *wR*2 = 0.076 for 773 observed reflections with *I* > 2σ(*I*). The silicate anion is based on pyrosilicate units of com­position [Si_2_O_7_]^6−^ with point-group symmetry *m* (*C_s_*). Charge com­pensation is achieved by the incorporation of rubidium and calcium cations distributed among a total of five independent sites within the asymmetric unit. Two of the nontetra­hedrally coordinated cation sites (*M*4 and *M*5) are exclusively occupied by calcium cations, which are surrounded by six O atoms in the form of octa­hedra or trigonal prisms, respectively. The rubidium cations on the *M*1–*M*3 sites show more com­plex coordination environments. The *M*2 site, for example, is characterized by a tricapped trigonal prism polyhedron. Notably, the *M*3 site exhibits a 50% population of Ca^2+^ and Rb^+^, respectively. The com­pound shows closer structural resemblances with K_2_Ca_2_Si_2_O_7_ and can be derived from a hexa­gonal aristotype with space-group symmetry *P*6_3_/*mmc* by displacements of the atoms. The corresponding distortion modes can be classified by certain irreducible representations of the high-symmetry parent phase. Structural investigations were com­pleted by determining the thermal expansion tensor for the tem­per­a­ture inter­val between −80 and 15 °C.

## Abstract 2

The crystal structure and thermal expansion of Rb_2_Ca_2_Si_2_O_7_ have been investigated from single-crystal diffraction data. The structural relationships with other pyrosilicates are discussed.

## Introduction

The ternary *A*_2_O–CaO–SiO_2_ systems, where *A* represents a chemical element in Group 1 of the Periodic Table have been the subject of many investigations in the past. However, the extent to which these systems were studied through synthesis experiments and/or thermodynamic modelling varies considerably depending on the specific alkali metal in question. This observation is directly correlated with the importance of a particular system for certain disciplines within the fields of inorganic chemistry and technical mineralogy. To date, the Na_2_O–CaO–SiO_2_ system has undoubtedly attracted the greatest attention (Morey & Bowen, 1925 ▸; Segnit, 1953 ▸; Williamson & Glasser, 1965 ▸; Shahid & Glasser, 1971 ▸; Zhang *et al.*, 2011 ▸; Santoso *et al.*, 2022 ▸). The corresponding melts have been of fundamental importance to the glass industry, facilitating the production of flat and hollow soda-lime silicate glass products that are ubiquitous in everyday life (Varshneya, 1994 ▸; Shelby, 2009 ▸). The crystalline counterparts cannot only occur as glass defects due to devitrification problems (Holland & Preston, 1938 ▸; Kahlenberg *et al.*, 2010 ▸), but have also been studied as optical diffusers (Butt *et al.*, 2014 ▸), constituents of bioactive ceramics (Reddy *et al.*, 2014 ▸; Zandi Karimi *et al.*, 2018 ▸) or as host materials for rare-earth-element-based silicate phosphors (Liu *et al.*, 2014 ▸; Parauha *et al.*, 2022 ▸). It is noteworthy that some sodium calcium silicates, such as combeite (Na_2_Ca_2_Si_3_O_9_), are also present in nature as exotic species in rather unusual petrological environments (Mitchell & Dawson, 2012 ▸; Weidendorfer *et al.*, 2016 ▸; Kahlenberg, 2023 ▸).

Conversely, the K_2_O–CaO–SiO_2_ system has only recently experienced a resurgence of inter­est, because several of the ternary phases can occur in ashes from biomass combustion and gasification (Olanders & Steenari, 1995 ▸; Chen & Zhao, 2016 ▸; Santoso *et al.*, 2020 ▸). During the last 15 years, eight potassium calcium silicates have been structurally characterized in detail, most of them for the first time. A recent summary of these phases, encom­passing a vast array of connectivities of the [SiO_4_] tetra­hedra silicate anions and new structure types, can be found in the article by Liu *et al.* (2021 ▸).

According to West (1978 ▸), four thermodynamically stable ternary phases occur in the Li_2_O–CaO–SiO_2_ system. A major subject of inter­est for lithium calcium silicates is their applications in glass ceramics (Al-Harbi, 2007 ▸) or for the synthesis of phosphors after doping with rare earth elements (Kim *et al.*, 2012 ▸; Wu *et al.*, 2020 ▸).

Because the highly radioactive and extremely unstable alkali metal francium is not a suitable subject for phase analytical studies, only two other systems remain for consideration, Rb_2_O–CaO–SiO_2_ and Cs_2_O–CaO–SiO_2_, both of which have been relegated to the backwaters of silicate research. One potential explanation for this fact is that these ternary silicate phases are expected to be more sensitive to water and humidity in com­parison to the corresponding com­pounds con­taining alkali elements with lower atomic numbers. Naturally, this aspect presents a clear disadvantage from an applicational perspective. For the rubidium-con­taining system, the existence of only one crystalline com­pound has been reported so far. Single crystals of Rb_2_Ca_2_Si_3_O_9_ were obtained using a polycrystalline precursor and an RbCl_2_ flux (Kahlenberg *et al.*, 2016 ▸). Its crystal structure is based on silicate anions forming *sechser* single chains.

In the course of a systematic investigation of the ternary Rb_2_O–CaO–SiO_2_ system, the crystal structure of a previously unknown com­pound, which was crystallized from the melt without the application of a mineralizer, is reported.

## Experimental

### Synthesis

The synthesis experiment for 1 g of a sample with an Rb_2_O:CaO:SiO_2_ molar ratio of 2:1:3 (or Rb_4_CaSi_3_O_9_) was based on a stoichiometric mixture of the following educts: Rb_2_CO_3_ (Aldrich, 99.8%), CaCO_3_ (calcite, Merck, >99.9%) and SiO_2_ (quartz, AlfaAesar, 99.995%). Prior to weighing on an analytical balance, the reagents were dried at 400 °C for a period of 24 h. In addition to removing physically adsorbed water, this step is important because rubidium carbonate is known to be very hygroscopic. Homogenization was per­formed with an agate mortar and a pestle for a duration of 15 min in a glove-bag under argon. The mixture was immediately transferred to a 50 ml platinum crucible, which was covered with a platinum lid. The con­tainer was heated in a box furnace in air from room tem­per­a­ture to 1050 °C at a rate of 6 °C min^−1^. The sample was annealed at the maximum tem­per­a­ture for 60 min and subsequently cooled to 800 °C at a rate of 0.3 °C min^−1^ before final quenching to ambient con­ditions. Weight losses were determined from weight differences before and after heating. The observed difference was 0.6% higher than the predicted value (based on CO_2_ release from the disintegration of the carbonates), indicating that losses due to Rb_2_O evaporation were small. A preliminary visual inspection of the product following the removal of the lid indicated that the sample had melted. The crucible was then stored in an evacuated desiccator for further analysis.

### Single-crystal diffraction

The solidified melt cake was mechanically separated from the crucible and further crushed in an agate mortar. Portions of the sample were immediately transferred to a glass slide into a drop of Paratone-N oil (Hampton Research) and in­ves­ti­gated under a polarizing binocular, which revealed the existence of transparent colourless birefringent single crystals (showing sharp extinction between crossed polarizers) up to 350 µm in size. The majority of the crystals were found to be at least partially embedded in an optically isotropic glassy matrix.

Notably, fresh transparent crystals when exposed to air at 38% relative humidity and 21 °C (laboratory conditions) on a glass slide began to become slightly opaque after 3 d. After 6 d, the samples were com­pletely opaque with a milky white colour, indicating an ongoing hydration reaction. However, the hydration product was not analysed further.

Several crystals displaying prismatic to plate-like mor­phol­ogy were isolated from the oil and affixed to glass fibers using fingernail hardener. They were subsequently studied using single-crystal diffraction performed on an Oxford Dif­fraction Gemini R Ultra diffractometer, which was equipped with a four-circle kappa goniometer and a Ruby CCD detector. Preliminary diffraction experiments were con­ducted with the objective of determining the unit-cell parameters. The screening process was performed in a dried air gas stream of −80 (2) °C generated by an Oxford Cryosystems Desktop Cooler to protect the samples from potential hydration. All crystals were found to belong to the same phase and exhibited an ortho­rhom­bic primitive metric that did not correspond to any entries of the Rb_2_O–CaO–SiO_2_ system or one of the relevant silicate subsystems currently available in the Inorganic Crystal Structure Database (Hellenbrandt, 2004 ▸). The sample with the best overall diffraction quality was selected for further structural analysis. A full sphere of reciprocal space up to 29.50° θ was obtained with Mo *K*α radiation (see Table 1 ▸). The data were processed using the *CrysAlis PRO* software package (Rigaku OD, 2020 ▸). Following indexing, the diffraction pattern was integrated. The data reduction process involved Lorentz and polarization corrections. Finally, the tem­per­a­ture was raised to 15 (2) °C and a second data collection was started using the identical run list employed for the low-tem­per­a­ture study, while keeping the crystal im­mer­sed in a dry environment (see Table 1 ▸). There was no evidence of a phase transition upon heating to ambient conditions. Once the correct chemical formula had been established on the basis of structure determination (see below), an analytical numeric absorption correction was applied to both data sets using a multifaceted crystal model.

The intensity statistics clearly indicated the presence of a centre of symmetry. Merging the two data sets in the ortho­rhom­bic Laue group 2/*m* 2/*m* 2/*m* resulted in reasonable inter­nal *R* values (see Table 1 ▸). Based on the observed reflection conditions (*hk*0): *h*+*k* = 2*n*, only the space groups *P*2_1_*mn*, *Pm*2_1_*n* and *Pmmn* remained. The structure solution for the low-tem­per­a­ture set was successfully initiated in the centrosymmetric space group using direct methods (*SIR2002*; Burla *et al.*, 2003 ▸), which provided a crystal-chemically sound starting model. One missing O atom was found from a difference Fourier map (*SHEXL97*; Sheldrick, 2008 ▸). The same software was also employed for subsequent full-matrix least-squares refinements. The scattering curves and anomalous dispersion coefficients were obtained from the *Inter­national Tables for Crystallography* (Vol. C; Prince, 2004 ▸). The final structure model obtained from the low-tem­per­a­ture data collection was then used as a starting point for the refinement of the structure under ambient conditions. The calculations with anisotropic displacement parameters for all atoms resulted in *R*1 residuals of 0.029 (at −80 °C) and 0.030 (at 15 °C) The largest shift/e.s.d. in the final cycles was < 0.001. Section 2.3 provides a detailed analysis of the site populations of the five non­tetra­hedrally coordinated cation sites in the asymmetric unit. The resulting chemical com­position from the structure analysis was Rb_2_Ca_2_Si_2_O_7_. Table 2 ▸ lists the final coordinates, site occupancies and equivalent isotropic displacement parameters, while Table 3 ▸ provides the anisotropic displacement parameters. Table 4 ▸ summarizes the selected inter­atomic distances. Structural features were illustrated using the *VESTA3* program (Momma & Izumi, 2011 ▸). Bond valence sum (BVS) calculations have been performed with the program *VaList* (Wills, 2010 ▸) using the parameter sets of Brown & Altermatt (1985 ▸) for Ca—O and Rb—O inter­actions, as well as Brese & O’Keeffe (1991 ▸) for the Si—O bonds. For the illustration of the three-dimensional representation surface of the thermal expansion tensor, the program *WinTensor* was employed (Kaminsky, 2014 ▸).

### Crystal structure

**Figure 1:**
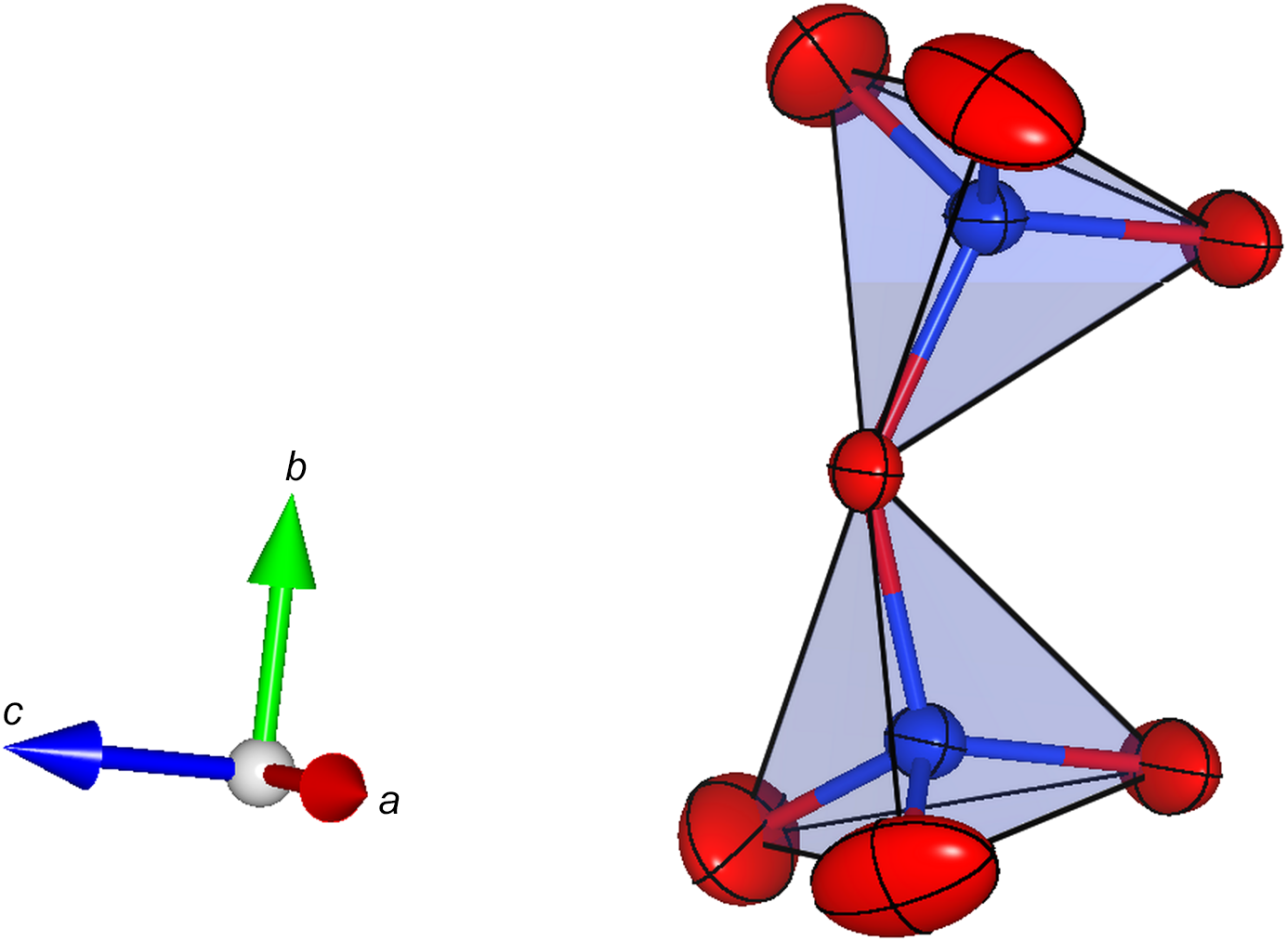
Side view of a single [Si_2_O_7_]^6−^ unit. Displacement ellipsoids are shown at the 80% probability level. Colour key: O atoms are red and Si atoms are blue.

The crystal structure is based on [Si_2_O_7_]^6−^ anions and can be classified as a sorosilicate (Liebau, 1985 ▸). The unit cell con­tains a total of two symmetrically independent bi­tetra­hedral units. Both silicate anions exhibit point-group symmetry *m* or *C_s_* (see Fig. 1 ▸). Charge com­pensation within the structure is achieved by monovalent rubidium and divalent calcium cations, which are distributed among a total of five different positions (*M*1–*M*5). Bond distance considerations indicated that *M*1 and *M*2 are pure rubidium sites, while *M*4 and *M*5 are occupied by calcium ions. Indeed, the results of the site-population refinements pointed to full occupancy with the respective two cation species. Conversely, *M*3 was identified as a mixed cation position, with a population of 51 (2)% Rb and 49 (2)% Ca. Therefore, we finally assumed a 1:1 ratio of rubidium and calcium on the *M*3 site, leading to a charge-neutral chemical com­position of Rb_2_Ca_2_Si_2_O_7_. Taking into account the initial com­position of the starting material, it can be concluded that the glass phase is enriched in Rb_2_O com­pared to the crystalline samples.

It is important to note that all geometrical parameters presented in this paragraph have been derived from the refinement of the data set collected at 15 °C. The Si—O bond distances within the two silicate dimers cover a considerable range, spanning from 1.591 (5) to 1.681 (2) Å. However, this variation aligns with the anti­cipated trends for [Si_2_O_7_]^6−^ groups com­prising one bridging and three terminal O atoms. The distances between the Si atoms and the terminal O atoms are notably shorter (average of both dimers = 1.599 Å) than the corresponding bond lengths to the bridging O atoms (average of the dimers = 1.680 Å). The observed shortening of the mean Si—O(terminal) bond distance by 0.081 Å can be attributed to the stronger attraction between the O and Si atoms than between the O atoms and the rubidium/calcium cations present in the structure. The distortion of the tetra­hedra is also reflected in the O—Si—O angles, which range from 102.8 (3) to 113.52 (14)°, respectively. Nevertheless, the mean O—Si—O angles are in close proximity to the ideal values for an ideal tetra­hedron (see Table 4 ▸). The degree of tetra­hedral distortion can be qu­anti­fied using the following two parameters: quadratic elongation (QE) and angle vari­ance (AV) (Robinson *et al.*, 1971 ▸). The numerical values for these parameters are also provided in Table 4 ▸. In fact, the distortions of both the crystallographically independent [Si_2_O_7_]^6−^ units are relatively minor, with the tetra­hedra around Si1 showing the least strain. Moreover, due to the point-group symmetry *m*, the silicate dimers display an eclipsed conformation. The Si—O—Si bond angles deviate from linearity, exhibiting significantly smaller values of 135.7 (4) and 139.4 (4)°. In particular, the second value is close to 140°, which is postulated to correspond to an unstrained Si—O—Si angle (Liebau, 1985 ▸).

**Figure 2:**
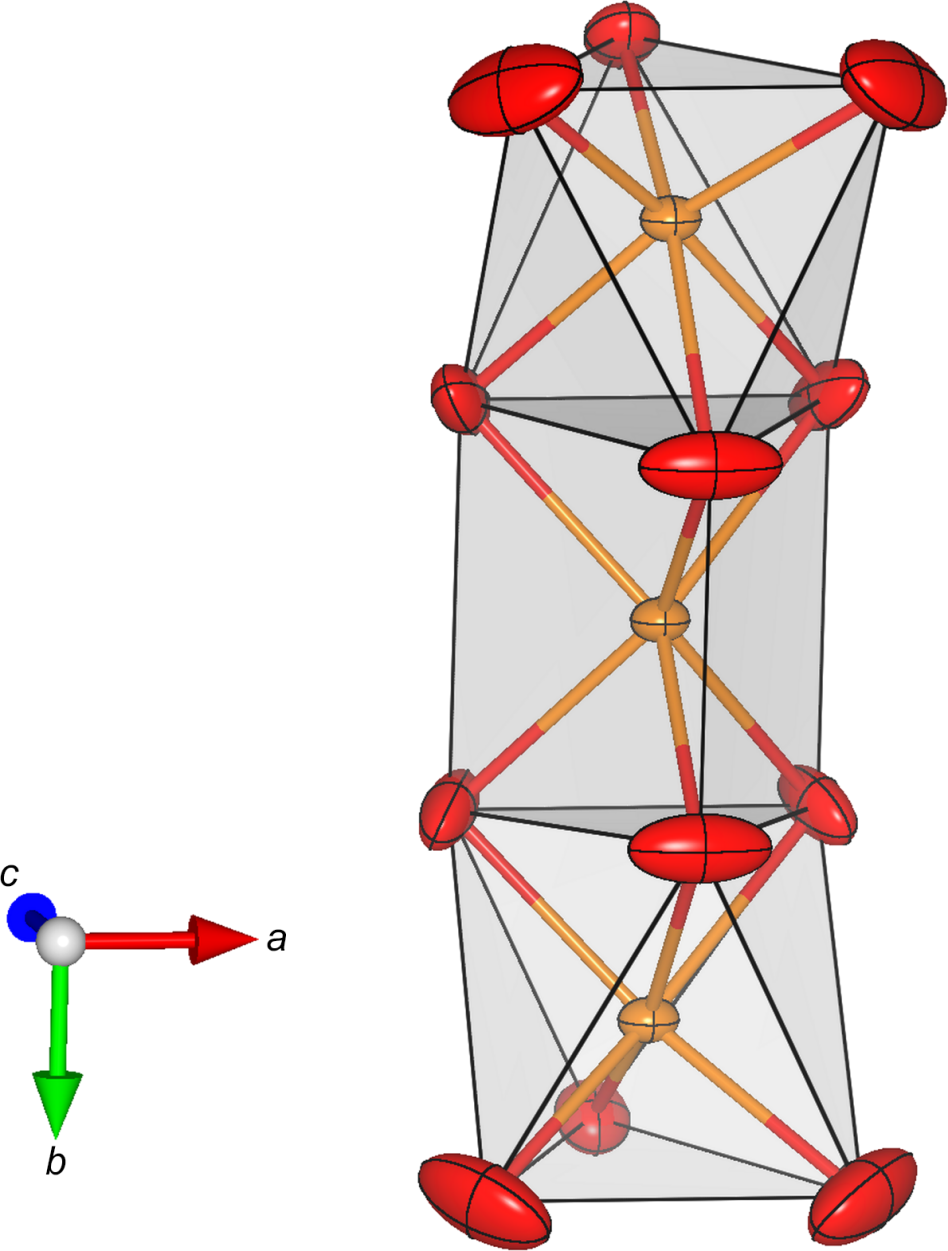
Side view of a single trimer con­taining two octa­hedra and a central trigonal prism. Displacement ellipsoids are drawn at the 80% probability level. Colour key: O atoms are red and Ca atoms are orange.

**Figure 3:**
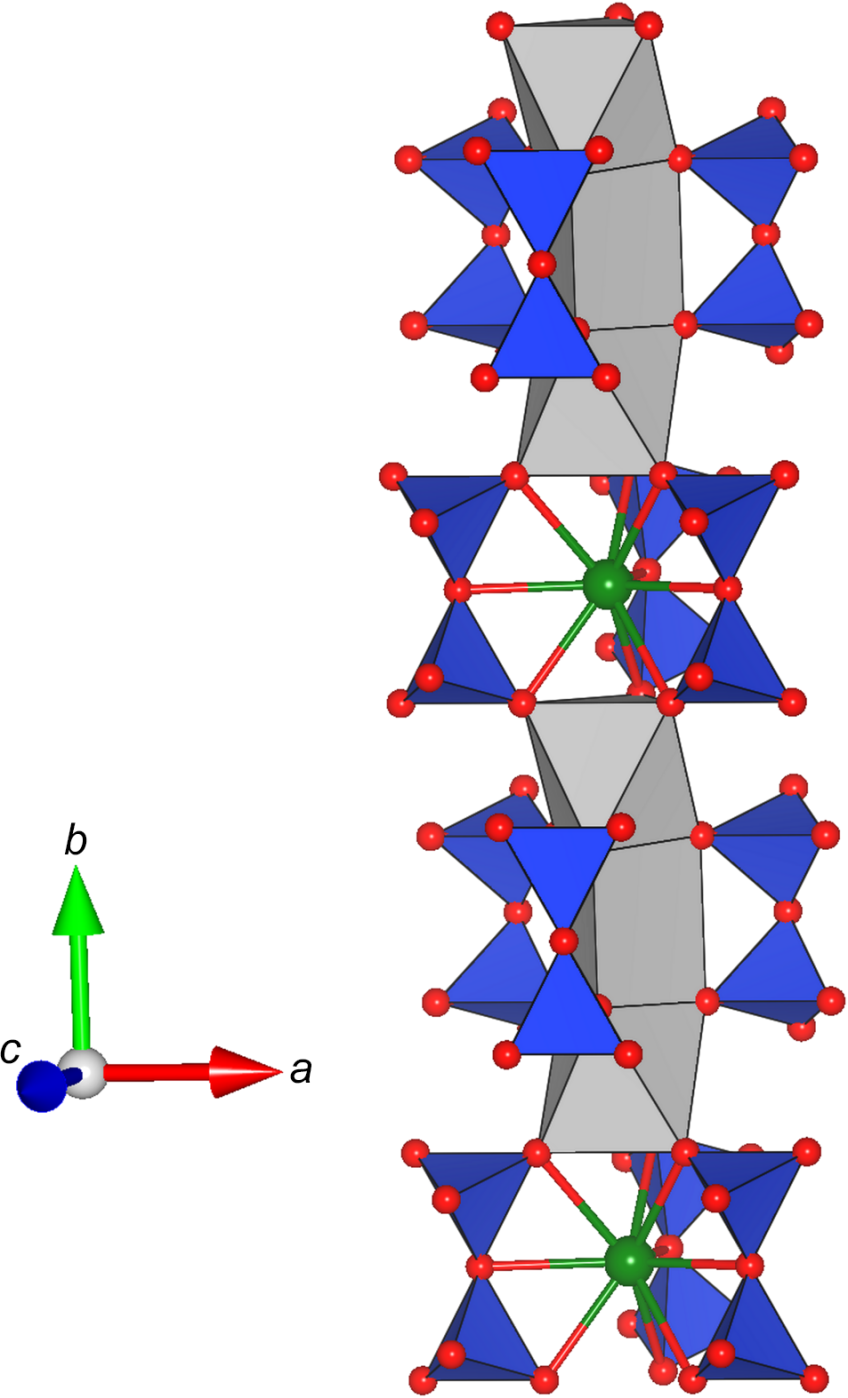
Side view of a single rod-like building element. Rb atoms occupying *M*2 are shown in green and are coordinated by nine O atoms in the form of a tricapped trigonal prism. O atoms are presented in red.

**Figure 4:**
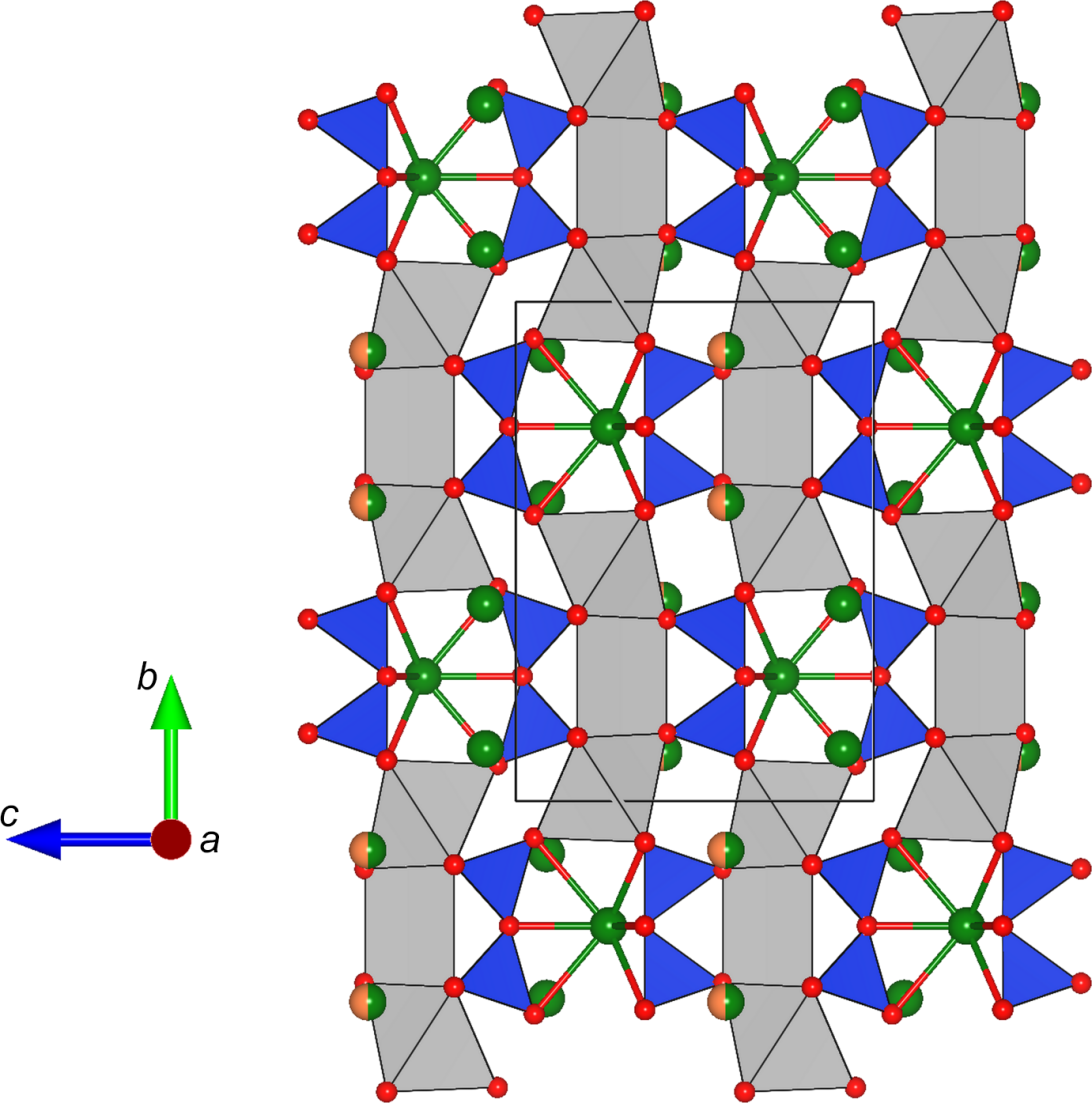
Projection of the whole crystal structure of Rb_2_Ca_2_Si_2_O_7_ along [100]. Potassium and calcium cations are illustrated in green and orange, respectively. O atoms are shown in red. The sizes of the two-coloured segments of the *M*3 site are the percentages determined from the site-occupancy refinements.

The Ca positions (*M*4 and *M*5) are coordinated by six oxygen ligands that form distorted octa­hedra (around *M*5) and trigonal prisms (around *M*4). Each trigonal prism shares opposing faces with two adjacent octa­hedra (see Fig. 2 ▸). The resulting tripolyhedral cluster has point-group symmetry *m*. Neighbouring clusters are linked by rubidium cations located at the *M*2 position. They are coordinated by nine O atoms, forming a highly elongated tricapped trigonal prism. Again, linkage is provided by shared faces, but this time between *M*5O_6_ and *M*2O_9_ polyhedra. Consequently, linear rod-like building blocks are obtained, that run parallel to the *b* axis (see Fig. 3 ▸). The [Si_2_O_7_]^6−^ groups serve to connect adjacent rods. Each dimer shares common oxygen anions with (i) a single trigonal prism around *M*4 and (ii) several surrounding *M*2O_9_ groups. Finally, the Rb and/or Ca atoms on the *M*1 and *M*3 positions com­plete the structure, occupying cavities above and below the silicate dimers. They are bonded to seven and eight O atoms, respectively, forming more irregular coordination polyhedra. A projection of the whole structure parallel to [100] is given in Fig. 4 ▸.

With the exception of the *M*3 site, the BVS calculations for the various cation positions yielded values that were close to the formal charges of the ions, assuming full occupancy with a single cation species: *M*1 1.129, *M*2 1.243, *M*4 1.942, *M*5 2.182, Si1 4.078 and Si2 4.019 (all data in valence units, v.u.). In the case of *M*3, however, a pronounced overbonding (1.778 v.u. for Rb) or underbonding (0.800 v.u. for Ca) was observed. This outcome provides further evidence for a mixed Rb/Ca occupancy. Notably, BVS calculations can permit an independent, though typically rather rough estimation, of the contents of two distinct atom types sharing the same position (Brown, 2016 ▸). The concentrations obtained using the corresponding bond-valence parameters in combination with the *M*3—O bond distances determined at 15 °C are as follows: 61% Rb and 39% Ca. This result is deemed to be in sufficiently good agreement with the percentages determined from the site-population refinements.

### Thermal expansion

**Figure 5:**
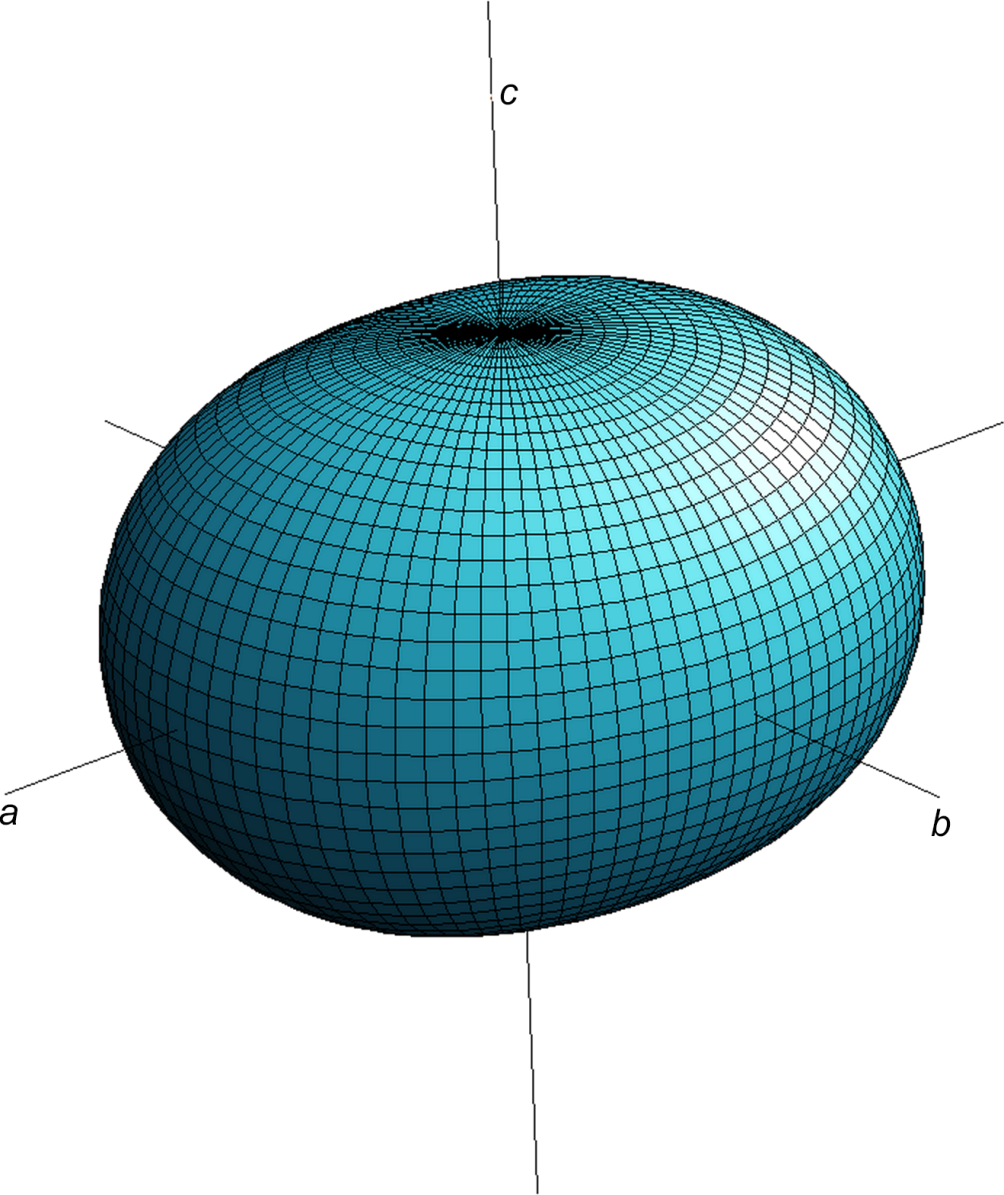
Three-dimensional representation surface of the average thermal expansion tensor of Rb_2_Ca_2_Si_2_O_7_ in the tem­per­a­ture inter­val between −80 and 15 °C.

The two sets of lattice parameters determined at −80 and 15 °C were employed to calculate the average thermal expansion tensor α_*ij*_ for the specified tem­per­a­ture inter­val from the thermal strain tensor ɛ_*ij*_ and the relationship 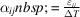. Due to the ortho­rhom­bic symmetry restrictions, the off-diagonal terms of the symmetric second-rank tensor ɛ_*ij*_ with *i* ≠ *j* must be strictly zero. The remaining three com­ponents can be obtained from the following expressions: ɛ_11_ = 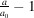, ɛ_22_ = 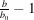 and ɛ_33_ = 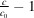. Notably, the lattice parameters with the suffix ‘zero’ pertain to the low-tem­per­a­ture data. In consequence, the thermal expansion tensor has the following form: 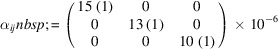


From the com­parison of the numerical values it is obvious that the thermal expansion is not extremely anisotropic. The largest (α_11_) and the smallest (α_33_) value differ by only a factor of 1.5. The expansion along [010], that is, along the rod-like building blocks of the crystal structure, is observed to have an inter­mediate value which is equivalent to the average of α_11_ and α_33_ within one standard deviation. By plotting the values of the thermal expansion tensor as a function of all directions one obtains a convenient geometric representation of the anisotropic behaviour of the tensor in the form of a surface in three-dimensional space (Fig. 5 ▸). The corresponding two-dimensional sections (**a**–**b**, **b**–**c** and **a**–**c**) are presented in Fig. S1 of the supporting information.

## Discussion

It is somewhat unexpected to find that the *M*3 site is occupied by both rubidium and calcium. Indeed, the two cations differ considerably concerning their ionic radii: *r*(Rb^+,[8]^) = 1.61 Å and *r*(Ca^2+,[8]^) = 1.12 Å (Shannon, 1976 ▸). In the only other structurally characterized rubidium calcium silicate, Rb_2_Ca_2_Si_3_O_9_, the two non­tetra­hedrally coordinated cation species are well ordered (Kahlenberg *et al.*, 2016 ▸). To exclude the possibility that the observation of a mixed population is an artifact due to an incorrect unit cell and/or symmetry, several additional tests were performed.

First, the frequency distributions of the experimentally determined Bragg peak positions when projected onto the *a*, *b* and *c* axes were com­puted and the corresponding maxima along these lines were visualized. Secondly, precession-type reconstructions of reciprocal space were calculated for the zero, first and second layers of reciprocal space for each of the three symmetry directions of the ortho­rhom­bic crystal system. Neither method yielded any indication of additional reflections requiring a larger cell. In other words, it can be excluded that our model corresponds to an average structure.

Finally, it was tested whether the unit cell was correct, but the structure was described in a symmetry that was too high. Indeed, a reduction in symmetry could allow for the possibility of cation ordering among the four symmetry-equivalent positions belonging to the Wyckoff position 4*e* of the *M*3 site. In the light of the aforementioned observed systematic absences, which clearly indicated the presence of an *n*-glide plane perpendicular to [001], the following *translationengleiche* subgroups of *Pmmn* were considered for a potential symmetry reduction: *P*2_1_*mn*, *P*112/*n*, *Pm*2_1_*n* and *P*11*n*. Notably, only a description in one of the latter two space groups involves a Wyckoff splitting of the 4*e* position, which is a prerequisite for cation ordering. Therefore, the model in *Pmmn* was transformed for each of the two relevant acentric subgroups and the refinement calculations were repeated. In both instances, instabilities of the refinements were recognized, which can be attributed to the presence of significant correlations between the coordinates and displacement parameters of those atom pairs, which were previously coupled by the centres of inversion present in *Pmmn*. Consequently, the refinements were restarted using adapted models in the low-symmetry space groups, wherein the atomic coordinates of all atoms except those of the former *M*3 site were constrained manually to conform to centrosymmetric structures. Despite the expected successful resolution of the correlation issue, the Rb and Ca ions demonstrated no tendency to order among the new sets of sites obtained from the Wyckoff splitting of the former *M*3 position. In conclusion, we found no evidence to suggest that the distribution of the rubidium and calcium on *M*3 is not statistically random.

The *c*/*a* ratio of the ortho­rhom­bic lattice parameters had a value close to 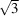, which is characteristic of an orthohexa­gonal cell. Although there is no doubt that the actual symmetry of Rb_2_Ca_2_Si_2_O_7_ is only ortho­rhom­bic, this observation prompted us to check for potential pseudosymmetry using the program *PSEUDO*, implemented in the Bilbao Crystallographic Server (Capillas *et al.*, 2011 ▸). Relative coordinates of all atoms were used, without distingushing between the various cation species present on the *M* sites. The search involving minimal supergroups was successful and indicated that the structure can be derived from an aristotype or the parent structure in *G* = *P*6_3_/*mmc* with *a*′ = 5.7352 and *c*′ = 13.8532 Å, provided that an inter­mediate step to *Z* = *Cmcm* is introduced and atomic shifts up to 1 Å are permitted. The 4 × 4 transformation matrix leading directly from parent structure to the structure in *H* = *Pmmn* is as follows: 
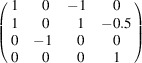


In the hexa­gonal aristotype, the sites *M*1 and *M*3, as well as *M*2 and *M*4, are symmetrically equivalent (Wyckoff positions 4*f* and 2*b* of *P*6_3_/*mmc*, respectively). *M*5 (in 2*a*) corresponds to a third non­tetra­hedrally coordinated cation position. Moreover, the asymmetric unit of the *P*6_3_/*mmc* structure con­tains a single Si atom (in 4*f*) and two O atoms (in 12*k* and 2*d*), that is, all [Si_2_O_7_] dimers in the unit cell are coupled by symmetry. Notably, the aristotype is isostructural with K_3_ScSi_2_O_7_ (Napper *et al.*, 2004 ▸). In this pyrosilicate, the Sc atoms occupy the octa­hedrally coordinated positions, while the potassium ions are located in the centres of the trigonal prisms.

With respect to the atomic coordinates, the symmetry break from the parent structure can be explained with the onset of several distortion modes, whose symmetry properties are given by the irreducible representations (irreps) of the space group *P*6_3_/*mmc* of the aristotype. The corresponding mode analysis was performed using the program *AMPLIMODES* (Orobengoa *et al.*, 2009 ▸). The observed symmetry reduction requires the irrep *M*_4_^−^ associated with the point *M* (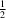 0 0) of the first Brillouin zone, as well as the zone-center irrep Γ_5_^+^. Furthermore, the fully symmetrical Γ_1_^+^ distortion is also allowed that retains the symmetry of the aristotype.

Using the crystallographic data, a reference structure was calculated in a first step representing the aristotype, but expressed in the low-symmetry space group *Pmmn*. From the com­parison of the reference structure with the actual model in *Pmmn* the resulting displacement field can be obtained, which is defined by the individual displacement vectors **u** for the atoms in the asymmetric unit of the reference structure. It defines the displacive distortions relating both structures. A detailed analysis of the shifts revealed that the O atoms are distinctly more affected. Their absolute values for the shifts vary between 0.345 and 0.649 Å. The Si atoms and most of the *M* sites show considerably smaller displacements. An exception, however, is the *M*5 position, which is displaced by about 0.147 Å. The average shift of all corresponding atom pairs in both structures has a value of 0.249 Å.

In the next step of mode analysis, the absolute amplitudes for the three individual com­ponents of the global distortions were calculated. Notably, the amplitudes were normalized with respect to the primitive unit cell of the high-symmetry structure. The relevant values are 1.61 (8) (for *M*_4_^−^), 0.147 (6) (for Γ_5_^+^) and 0.02 (8) Å (for Γ_1_^+^), indicating that the onset of the *M*_4_^−^ distortion triggers the symmetry break. Finally, for each involved irrep the corresponding polarization vector was obtained. The actual distortion of a specific mode can then be obtained by multiplying the com­ponents of the polarization vector with the amplitudes mentioned above. In order to obtain a concise graphical overview of the distortion fields, individual displacements of the most affected atoms O1 to O5 belonging to the [Si_2_O_7_]^6−^ groups calculated for the dominant *M*_4_^−^ representation only have been visualized using the program *VESTA3* (see Fig. S2 in the supporting information).

**Figure 6:**
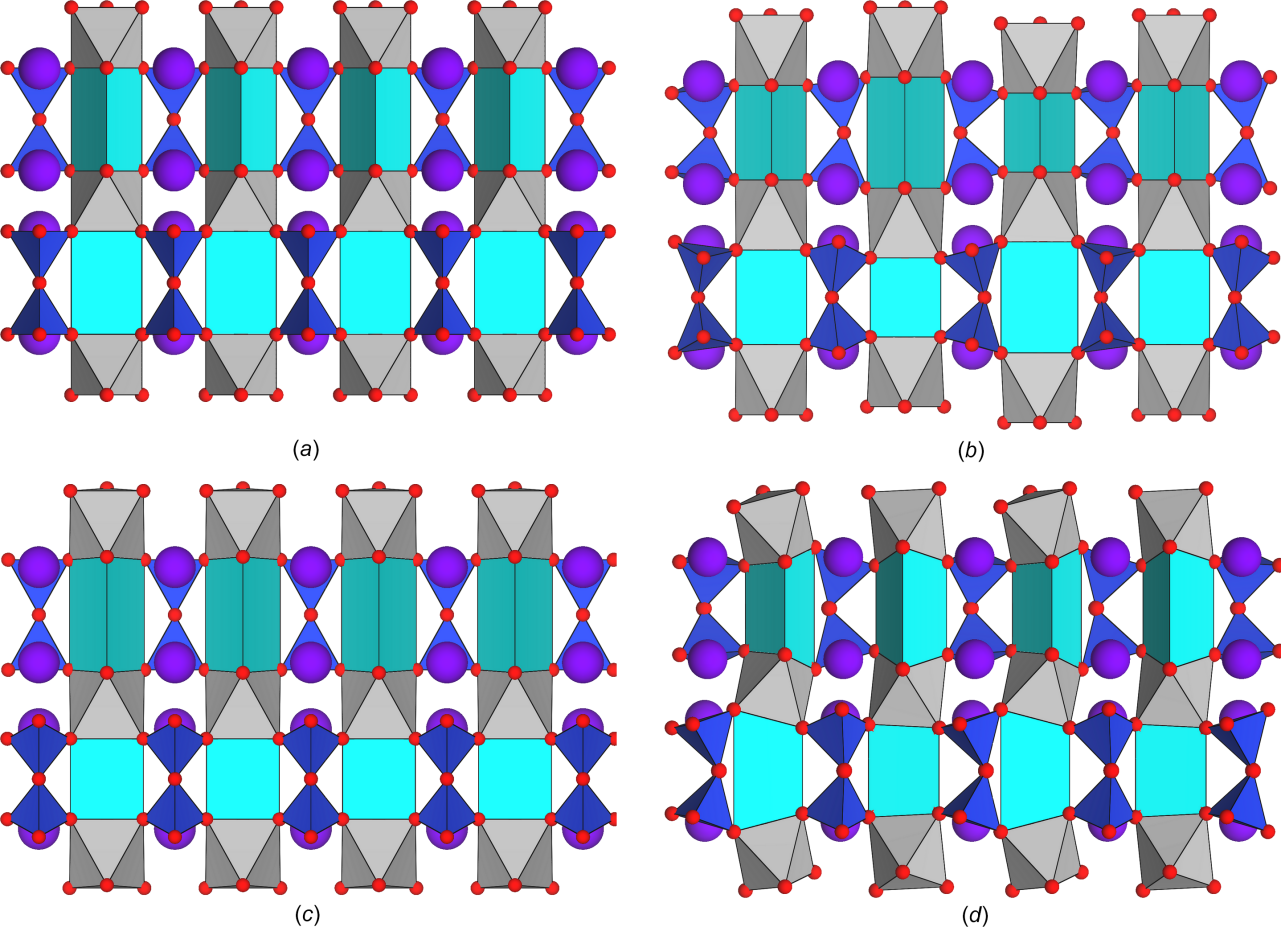
Structural differences within a 6.5 Å wide slab con­taining a sequence of four rods between (*a*) the aristotype, view along [120] (*P*6_3_/*mmc*, *a* = 5.6065 and *c* = 13.6420 Å), (*b*) K_2_Ca_2_Si_2_O_7_, view along [110] (*P*6_3_/*m*, *a* = 9.8020 and *c* = 13.8781 Å), (*c*) Rb_2_Ca_2_Si_2_O_7_, view along [001] (*Pmmn*, *a* = 5.7363, *b* = 13.8532 and *c* = 9.9330 Å), and (*d*) Na_3_ScSi_2_O_7_, view along [1
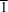
0] (*Pbnm*, *a* = 5.3540, *b* = 9.3470 and *c* = 13.0890 Å). Different colours simply highlight the various coordination polyhedra, but do not provide any information about their occupation with different cation species.

To date, four alkali alkaline-earth silicates with the general formula *A*_2_Ca_2_Si_2_O_7_ have been the subject of structural investigations. With the exception of Na_2_Ca_2_Si_2_O_7_, which is a mixed anion silicate con­taining insular [SiO_4_] tetra­hedra and [Si_3_O_10_] trimers in a 1:1 ratio (Kahlenberg & Hösch 2002 ▸), the corresponding lithium, potassium and rubidium com­pounds are characterized by [Si_2_O_7_]^6−^ units. Li_2_Ca_2_Si_2_O_7_ represents a unique structure type (Kahlenberg *et al.*, 2015 ▸) and can be rationalized as a three-dimensional framework structure based on corner-sharing [LiO_4_] and [SiO_4_] tetra­hedra, in­cluding an O^[3]^-type bridging oxygen node linking three adjacent tetra­hedra. While the lithium calcium disilicate and the com­pound under investigation exhibit no closer structural relationship, K_2_Ca_2_Si_2_O_7_ (space group *P*6_3_/*m*) and Rb_2_Ca_2_Si_2_O_7_ can be derived from the condensation of the same type of rod-like elements that have been introduced in Section 2.3. In more detail, both com­pounds can be considered as derivative structures of the same aristotype. However, the distortion patterns resulting in the hexa­gonal potassium and the ortho­rhom­bic rubidium phase are different. Fig. 6 ▸ illustrates the differences between the parent structure, the two above-mentioned less-symmetric alkali alkaline-earth silicates, as well as related Na_3_ScSi_2_O_7_ (Skrzat *et al.*, 1969 ▸), by showing a 6.5 Å wide slab for each structure that con­tains a sequence of four consecutive rods linked by [Si_2_O_7_]^6−^ units. For the sake of clarity, the structures have been simplified slightly. In fact, some of the coordination polyhedra are shown as distorted trigonal prisms, although in some cases there are additional O atoms capping two or three of the prismatic faces that actually do belong to the coordination sphere.

## Conclusion

The current crystal structure is an inter­esting example of the substitution of two cation species with very different ionic radii. For several potassium–calcium silicates, including K_2_Ca_2_Si_2_O_7_, the replacement of calcium by the substanti­ally larger potassium ion has been reported previously. However, the evidence in these cases was based solely on bond-valence calculations, as the two cation types are isoelectronic with 19 electrons each and thus cannot be discriminated from each other using direct site-population refinements by X-ray diffraction data. Conversely, in the case of Rb_2_Ca_2_Si_2_O_7_, the ion types differ significantly from each other in terms of the number of electrons, thereby rendering diffraction methods a viable additional evidence for a substitution of Rb with Ca. It is noteworthy that the discrepancy between the respective values of the radii for eight-coordinated potassium and rubidium is a mere 6% (Shannon, 1976 ▸).

As mentioned in the *Introduction*, the crystalline com­pounds of the rubidium–calcium and caesium–calcium silicate groups have been studied only rudimentarily, if at all. This opens up new possibilities for systematic crystal chemical investigations of the influence of the size of the alkali cations on the formation of certain structure types. As Liebau (1985 ▸) demonstrated in his seminal book on oxosilicates, correlations between the radii of the non­tetra­hedrally coordinated cations and various structural aspects, including, among others, ring sizes or chain periodicities in phyllo- and inosilicates, could be deciphered. However, this requires that the data set under consideration con­tains a sufficiently large number of representatives. Of particular inter­est, of course, are com­pounds with equal proportions of the various monovalent, divalent and tetra­valent cation types in the formula unit, such as the family of *A*_2_Ca_2_Si_2_O_7_ com­pounds. In order to obtain a com­prehensive crystallographic understanding of the com­pounds belonging to this general com­position, it is necessary to ascertain the structure of the missing Cs phase. This objective is currently being pursued through synthesis experiments.

The present work is a first contribution to a project that will investigate structural relationships systematically and at the same time provide new information on phase equilibria in the Rb_2_O–CaO–SiO_2_ and Cs_2_O–CaO–SiO_2_ systems.

## Supplementary Material

Crystal structure: contains datablock(s) RT, LT, global. DOI: 10.1107/S2053229625001196/ef3065sup1.cif

Structure factors: contains datablock(s) RT. DOI: 10.1107/S2053229625001196/ef3065RTsup2.hkl

Structure factors: contains datablock(s) LT. DOI: 10.1107/S2053229625001196/ef3065LTsup3.hkl

Supporting information file. DOI: 10.1107/S2053229625001196/ef3065sup4.pdf

CCDC references: 2422698, 2422697

## Figures and Tables

**Table 1:** Experimental details For both determinations: Rb_2_Ca_2_Si_2_O_7_, *M*_r_ = 419.28, orthorhombic, *Pmmn*, *Z* = 4. Experiments were carried out with Mo *K*α radiation using a Rigaku Gemini R Ultra diffractometer equipped with a four-circle kappa goniometer and a Ruby CCD detector. The absorption correction was analytical [*CrysAlis PRO* (Rigaku OD, 2020 ▸), based on expressions derived by Clark & Reid (1995 ▸)]. Refinement was on 78 parameters.

	**Data at 15 °C (RT)**	**Data at −80 °C (LT)**
Crystal data		
Temperature (K)	288	193
*a*, *b*, *c* (Å)	5.7363 (6), 13.8532 (12), 9.933 (1)	5.7281 (6), 13.8361 (13), 9.9233 (11)
*V* (Å^3^)	789.34 (13)	786.47 (14)
μ (mm^−1^)	14	14.05
Crystal size (mm)	0.32 × 0.1 × 0.04	0.32 × 0.1 × 0.04

*T*_min_, *T*_max_	0.121, 0.678	0.109, 0.675
No. of measured, independent and observed [*I* > 2σ(*I*)] reflections	10834, 931, 773	10870, 929, 777
*R*_int_	0.058	0.059
(sin θ/λ)_max_ (Å^−1^)	0.625	0.625

Refinement		
*R*[*F*^2^ > 2σ(*F*^2^)], *wR*(*F*^2^), *S*	0.030, 0.076, 1.06	0.029, 0.067, 1.07
No. of reflections	931	929
Δρ_max_, Δρ_min_ (e Å^−3^)	0.86, −0.70	0.93, −0.75

Computer programs: *CrysAlis PRO* (Rigaku OD, 2020 ▸), *SIR2002* (Burla *et al.*, 2003 ▸) and *SHELXL97* (Sheldrick, 2008 ▸).

**Table 2:** Atomic coordinates (×10^4^, origin choice 2 of space group *Pmmn*) and equivalent isotropic displacement parameters (Å^2^ × 10^3^) for Rb_2_Ca_2_Si_2_O_7_ First line 15 °C and second line −80 °C. *U*_eq_ is defined as one third of the trace of the orthogonalized *U_ij_* tensor. *M*1 and *M*2 are exclusively occupied by rubidium, while *M*4 and *M*5 represent pure Ca sites. *M*3 is a mixed Rb–Ca position, with a population of 50% rubidium and 50% calcium.

Atom	Wyckoff site	Site symmetry	*x*	*y*	*z*	*U*_eq_
*M*1	4*e*	*m*..	7500	1049 (1)	854 (1)	18 (1)
			7500	1048 (1)	853 (1)	14 (1)
*M*2	2*a*	*mm*2	2500	2500	2585 (1)	16 (1)
			2500	2500	2588 (1)	12 (1)
*M*3	4*e*	*m*..	2500	980 (1)	5869 (1)	21 (1)
			2500	980 (1)	5869 (1)	21 (1)
*M*4	2*b*	*mm*2	7500	2500	7494 (2)	10 (1)
			7500	2500	7492 (2)	8 (1)
*M*5	4*e*	*m*..	2500	5106 (1)	2489 (1)	10 (1)
			2500	5108 (1)	2489 (1)	9 (1)
Si1	4*e*	*m*..	7500	1362 (1)	4187 (2)	15 (1)
			7500	1362 (1)	4186 (2)	14 (1)
Si2	4*e*	*m*..	2500	1396 (1)	9193 (2)	9 (1)
			2500	1396 (1)	9192 (2)	8 (1)
O1	8*g*	1	181 (5)	1274 (2)	8280 (3)	23 (1)
			183 (5)	1274 (2)	8280 (3)	21 (1)
O2	4*e*	*m*..	2500	734 (3)	10515 (4)	19 (1)
			2500	732 (3)	10520 (4)	16 (1)
O3	2*a*	*mm*2	2500	2500	9820 (7)	26 (2)
			2500	2500	9825 (6)	22 (2)
O4	8*g*	1	5246 (7)	822 (3)	3605 (4)	44 (1)
			5242 (7)	821 (3)	3604 (4)	43 (1)
O5	2*b*	*mm*2	7500	2500	3599 (6)	14 (1)
			7500	2500	3598 (6)	12 (1)
O6	4*e*	*m*..	7500	1355 (3)	5789 (4)	35 (1)
			7500	1361 (3)	5793 (5)	32 (1)

**Table 3:** Anisotropic displacement parameters (Å^2^ × 10^3^) for Rb_2_Ca_2_Si_2_O_7_ The anisotropic displacement factor exponent takes the form: −2π^2^[*h*^2^a*^2^*U*_11_ + ⋯ + 2hka*b*U_12_]. First line 15 °C and second line −80 °C.

	*U*_11_	*U*_22_	*U*_33_	*U*_23_	*U*_13_	*U*_12_
*M*1	14 (1)	26 (1)	13 (1)	0 (1)	0	0
	11 (1)	20 (1)	10 (1)	0 (1)	0	0
*M*2	18 (1)	14 (1)	16 (1)	0	0	0
	14 (1)	10 (1)	13 (1)	0	0	0
*M*3	18 (1)	41 (1)	13 (1)	−7 (1)	0	0
	16 (1)	35 (1)	11 (1)	−7 (1)	0	0
*M*4	13 (1)	7 (1)	11 (1)	0	0	0
	10 (1)	5 (1)	9 (1)	0	0	0
*M*5	13 (1)	7 (1)	10 (1)	0 (1)	0	0
	12 (1)	6 (1)	9 (1)	0 (1)	0	0
Si1	22 (1)	11 (1)	12 (1)	0 (1)	0	0
	21 (1)	10 (1)	11 (1)	0 (1)	0	0
Si2	9 (1)	7 (1)	12 (1)	2(1)	0	0
	7 (1)	6 (1)	11 (1)	2(1)	0	0
O1	17 (2)	21 (2)	32 (2)	7 (1)	−11 (1)	−7 (1)
	14 (2)	19 (2)	31 (2)	8(1)	−11 (1)	−7 (1)
O2	20 (2)	18 (2)	19 (2)	7(2)	0	0
	17 (2)	14 (2)	19 (2)	6(2)	0	0
O3	43 (4)	15 (3)	21 (3)	0	0	0
	40 (4)	10 (3)	15 (3)	0	0	0
O4	53 (3)	32 (2)	46 (2)	5 (2)	−25 (2)	−18 (2)
	50 (3)	28 (2)	50 (2)	5 (2)	−25 (2)	−16 (2)
O5	21 (3)	13 (3)	9 (3)	0	0	0
	20 (3)	10 (3)	6 (3)	0	0	0
O6	76 (4)	17 (2)	11 (2)	0(2)	0	0
	70 (4)	12 (2)	15 (2)	−1 (2)	0	0

**Table 4:** Selected bond lengths up to 3.2 Å and bond angles (°) for Rb_2_Ca_2_Si_2_O_7_ For the tetra­hedra and octa­hedra, the distortion parameters QE (quadratic elongation) and AV (angle variance) have been calculated.

15 °C		−80 °C	
*M*1—O2^i^	2.819 (4)	*M*1—O2^i^	2.815 (4)
*M*1—O2^ii^	2.9206 (9)	*M*1—O2^ii^	2.9160 (9)
*M*1—O2^iii^	2.9206 (9)	*M*1—O2^iii^	2.9160 (9)
*M*1—O1^iv^	2.999 (3)	*M*1—O1^iv^	2.997 (3)
*M*1—O1^iii^	2.999 (3)	*M*1—O1^iii^	2.997 (3)
*M*1—O4^v^	3.039 (4)	*M*1—O4	3.037 (4)
*M*1—O4	3.039 (4)	*M*1—O4^v^	3.037 (4)
<*M*1—O>	2.962	<*M*1—O>	2.959
*M*2—O3^ii^	2.746 (7)	*M*2—O3^ii^	2.742 (6)
*M*2—O4^vi^	2.985 (4)	*M*2—O4^vi^	2.980 (4)
*M*2—O4^vii^	2.985 (4)	*M*2—O4^vii^	2.980 (4)
*M*2—O4^viii^	2.985 (4)	*M*2—O4	2.980 (4)
*M*2—O4	2.985 (4)	*M*2—O4^viii^	2.980 (4)
*M*2—O5	3.0401 (19)	*M*2—O5	3.0343 (19)
*M*2—O5^ix^	3.0401 (19)	*M*2—O5^ix^	3.0343 (19)
*M*2—O2^ii^	3.196 (4)	*M*2—O2^ii^	3.193 (4)
*M*2—O2^*x*^	3.196 (4)	*M*2—O2^*x*^	3.193 (4)
<*M*2—O>	3.018	<*M*2—O>	3.013
*M*3—O4^viii^	2.754 (5)	*M*3—O4^vii^	2.752 (5)
*M*3—O4	2.754 (5)	*M*3—O4	2.752 (5)
*M*3—O1^viii^	2.770 (4)	*M*3—O1^vii^	2.765 (4)
*M*3—O1	2.770 (4)	*M*3—O1	2.765 (4)
*M*3—O4^xii^	2.860 (4)	*M*3—O4^xii^	2.855 (4)
*M*3—O4^i^	2.860 (4)	*M*3—O4^i^	2.855 (4)
*M*3—O6	2.9160 (9)	*M*3—O6	2.9131 (9)
*M*3—O6^ix^	2.9160 (9)	*M*3—O6^ix^	2.9131 (9)
<*M*3—O>	2.825	<*M*3—O>	2.821
*M*4—O6	2.320 (5)	*M*4—O6	2.308 (5)
*M*4—O6^xi^	2.320 (5)	*M*4—O6^xi^	2.308 (5)
*M*4—O1^xiii^	2.421 (3)	*M*4—O1^xiii^	2.419 (3)
*M*4—O1^vii^	2.421 (3)	*M*4—O1^vii^	2.419 (3)
*M*4—O1^viii^	2.421 (3)	*M*4—O1^xiv^	2.419 (3)
*M*4—O1^xiv^	2.421 (3)	*M*4—O1^viii^	2.419 (3)
<*M*4—O>	2.387	<*M*4—O>	2.382
*M*5—O2^*x*^	2.279 (4)	*M*5—O2^*x*^	2.274 (4)
*M*5—O4^vii^	2.317 (4)	*M*5—O4^viii^	2.312 (4)
*M*5—O4^vi^	2.317 (4)	*M*5—O4^vi^	2.312 (4)
*M*5—O1^xix^	2.359 (3)	*M*5—O1^xix^	2.355 (3)
*M*5—O1^xx^	2.359 (3)	*M*5—O1^xx^	2.355 (3)
*M*5—O6^xxi^	2.435 (5)	*M*5—O6^xxi^	2.431 (5)
<*M*5—O>	2.345	<*M*5—O>	2.340
QE = 1.020	AV = 69.94	QE = 1.020	AV = 70.19
Si1—O6	1.591 (5)	Si1—O6	1.595 (5)
Si1—O4	1.601 (4)	Si1—O4	1.602 (4)
Si1—O4^v^	1.601 (4)	Si1—O4^v^	1.602 (4)
Si1—O5	1.681 (2)	Si1—O5	1.679 (2)
<Si1—O>	1.618	<Si1—O>	1.620
QE = 1.001	AV = 2.51	QE = 1.001	AV = 2.48
Si2—O2	1.602 (4)	Si2—O2	1.606 (4)
Si2—O1	1.619 (3)	Si2—O1	1.615 (3)
Si2—O1^viii^	1.619 (3)	Si2—O1^vii^	1.615 (3)
Si2—O3	1.651 (3)	Si2—O3	1.652 (3)
<Si2—O>	1.622	<Si2—O>	1.622
QE = 1.004	AV = 16.51	QE = 1.004	AV = 17.44
			
O—Ca—O angles			
O6—*M*4—O6^xi^	86.2 (2)	O6—*M*4—O6^xi^	86.2 (2)
O6—*M*4—O1^xiii^	135.66 (10)	O6—*M*4—O1^xiii^	135.64 (10)
O6^xi^—*M*4—O1^xiii^	75.89 (12)	O6^xi^—*M*4—O1^xiii^	75.93 (12)
O6—*M*4—O1^vii^	135.66 (10)	O6—*M*4—O1^vii^	75.93 (12)
O6^xi^—*M*4—O1^vii^	75.89 (12)	O6^xi^—*M*4—O1^vii^	135.64 (10)
O1^xiii^—*M*4—O1^vii^	78.88 (14)	O1^xiii^—*M*4—O1^vii^	142.31 (18)
O6—*M*4—O1^viii^	75.89 (12)	O6—*M*4—O1^xiv^	75.93 (12)
O6^xi^—*M*4—O1^viii^	135.66 (10)	O6^xi^—*M*4—O1^xiv^	135.64 (10)
O1^xiii^—*M*4—O1^viii^	142.33 (18)	O1^xiii^—*M*4—O1^xiv^	89.08 (14)
O1^vii^—*M*4—O1^viii^	89.11 (14)	O1^vii^—*M*4—O1^xiv^	78.90 (14)
O6—*M*4—O1^xiv^	75.89 (12)	O6—*M*4—O1^viii^	135.64 (10)
O6^xi^—*M*4—O1^xiv^	135.66 (10)	O6^xi^—*M*4—O1^viii^	75.93 (12)
O1^xiii^—*M*4—O1^xiv^	89.11 (14)	O1^xiii^—*M*4—O1^viii^	78.90 (14)
O1^vii^—*M*4—O1^xiv^	142.33 (18)	O1^vii^—*M*4—O1^viii^	89.08 (14)
O1^viii^—*M*4—O1^xiv^	78.88 (14)	O1^xiv^—*M*4—O1^viii^	142.31 (18)
			
O2^x^—*M*5—O4^vii^	97.40 (13)	O2^x^—*M*5—O4^viii^	97.30 (13)
O2^x^—*M*5—O4^vi^	97.40 (13)	O2^x^—*M*5—O4^vi^	97.30 (13)
O4^vii^—*M*5—O4^vi^	85.7 (2)	O4^viii^—*M*5—O4^vi^	85.6 (2)
O2^x^—*M*5—O1^xix^	94.14 (12)	O2^x^—*M*5—O1^xix^	94.14 (12)
O4^vii^—*M*5—O1^xix^	168.20 (13)	O4^viii^—*M*5—O1^xix^	168.32 (13)
O4^vi^—*M*5—O1^xix^	95.30 (14)	O4^vi^—*M*5—O1^xix^	95.29 (14)
O2^x^—*M*5—O1^xx^	94.14 (12)	O2^x^—*M*5—O1^xx^	94.14 (12)
O4^vii^—*M*5—O1^xx^	95.30 (14)	O4^viii^—*M*5—O1^xx^	95.29 (14)
O4^vi^—*M*5—O1^xx^	168.20 (13)	O4^vi^—*M*5—O1^xx^	168.32 (13)
O1^xix^—*M*5—O1^xx^	81.37 (16)	O1^xix^—*M*5—O1^xx^	81.50 (16)
O2^x^—*M*5—O6^xxi^	165.38 (16)	O2^x^—*M*5—O6^xxi^	165.29 (16)
O4^vii^—*M*5—O6^xxi^	93.30 (13)	O4^viii^—*M*5—O6^xxi^	93.48 (13)
O4^vi^—*M*5—O6^xxi^	93.30 (13)	O4^vi^—*M*5—O6^xxi^	93.48 (13)
O1^xix^—*M*5—O6^xxi^	74.91 (11)	O1^xix^—*M*5—O6^xxi^	74.85 (11)
O1^xx^—*M*5—O6^xxi^	74.91 (11)	O1^xx^—*M*5—O6^xxi^	74.85 (11)
			
O—Si—O angles			
O6—Si1—O4	110.99 (19)	O6—Si1—O4	111.10 (19)
O6—Si1—O4^v^	110.99 (19)	O6—Si1—O4^v^	111.10 (19)
O4—Si1—O4^v^	107.7 (3)	O4—Si1—O4^v^	107.6 (3)
O6—Si1—O5	110.6 (3)	O6—Si1—O5	110.4 (3)
O4—Si1—O5	108.21 (18)	O4—Si1—O5	108.26 (18)
O4^v^—Si1—O5	108.21 (18)	O4^v^—Si1—O5	108.26 (18)
<O—Si1—O>	109.45	<O—Si1—O>	109.45
			
O2—Si2—O1	113.52 (14)	O2—Si2—O1	113.58 (14)
O2—Si2—O1^viii^	113.52 (14)	O2—Si2—O1^vii^	113.58 (14)
O1—Si2—O1^viii^	110.5 (3)	O1—Si2—O1^vii^	110.5 (3)
O2—Si2—O3	102.8 (3)	O2—Si2—O3	102.5 (3)
O1—Si2—O3	107.96 (17)	O1—Si2—O3	108.05 (16)
O1^viii^—Si2—O3	107.96 (17)	O1^vii^—Si2—O3	108.05 (17)
<O—Si2—O>	109.38	<O—Si2—O>	109.38
			
Si—O—Si angles			
Si2^vii^—O3—Si2	135.7 (4)	Si2—O3—Si2^viii^	135.3 (4)
Si1^xi^—O5—Si1	139.4 (4)	Si1^xi^—O5—Si1	139.3 (4)

Symmetry codes: (i) −*x* + 1, −*y*, −*z* + 1; (ii) *x*, *y*, *z* − 1; (iii) *x* + 1, *y*, *z* − 1; (iv) −*x* + 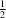, *y*, *z* − 1; (v) −*x* + 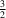, *y*, *z*; (vi) *x*, −*y* + 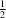, *z*; (vii) −*x* + 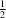, −*y* + 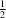, *z*; (viii) −*x* + 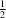, *y*, *z*; (ix) *x* − 1, *y*, *z*; (x) −*x* + 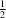, −*y* + 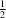, *z* − 1; (xi) −*x* + 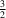, −*y* + 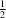, *z*; (xii) *x* − 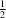, −*y*, −*z* + 1; (xiii) *x* + 1, −*y* + 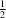, *z*; (xiv) *x* + 1, *y*, *z*; (xix) *x* + 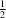, *y* + 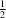, −*z* + 1; (xx) −*x*, *y* + 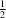, −*z* + 1; (xxi) *x* − 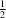, *y* + 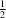, −*z* + 1.

## References

[bb1] Al-Harbi, O. A. (2007). *Eur. J. Glass. Sci. Technol.* **A48**, 35–40.

[bb2] Brese, N. E. & O’Keeffe, M. (1991). *Acta Cryst.* B**47**, 192–197.

[bb3] Brown, I. D. (2016). *The Chemical bond in Inorganic Chemistry: The Bond Valence Model*, 2nd ed., p. 315. Oxford University Press.

[bb4] Brown, I. D. & Altermatt, D. (1985). *Acta Cryst.* B**41**, 244–247.

[bb5] Burla, M. C., Camalli, M., Carrozzini, B., Cascarano, G. L., Giacovazzo, C., Polidori, G. & Spagna, R. (2003). *J. Appl. Cryst.* **36**, 1103. 10.1107/s0108767399007205 10927316

[bb6] Butt, H., Knowles, K. M., Montelongo, Y., Amaratunga, G. A. J. & Wilkinson, T. D. (2014). *ACS Nano*, **8**, 2929–2935. 10.1021/nn500155e 24559189

[bb7] Capillas, C., Tasci, E. S., de la Flor, G., Orobengoa, D., Perez-Mato, J. M. & Aroyo, M. I. (2011). *Z. Kristallogr.* **226**, 186–196.

[bb8] Chen, M. & Zhao, B. (2016). *Fuel*, **180**, 638–644.

[bb9] Clark, R. C. & Reid, J. S. (1995). *Acta Cryst.* A**51**, 887–897.

[bb10] Hellenbrandt, M. (2004). *Crystallogr. Rev.* **10**, 17–22.

[bb11] Holland, A. J. & Preston, E. (1938). *J. Soc. Glass Technol.* **21**, 395–408.

[bb12] Kahlenberg, V. (2023). *Miner. Petrol.* **117**, 293–306.

[bb13] Kahlenberg, V., Brunello, E., Hejny, C., Krüger, H., Schmidmair, D., Tribus, M. & Többens, D. (2015). *J. Solid State Chem.* **225**, 155–167.

[bb14] Kahlenberg, V., Girtler, D., Arroyabe, E., Kaindl, R. & Többens, D. (2010). *Miner. Petrol.* **100**, 1–9.

[bb15] Kahlenberg, V. & Hösch, A. (2002). *Z. Kristallogr.* **217**, 155–163.

[bb16] Kahlenberg, V., Müllner, M., Schmidmair, D., Perfler, L. & Többens, D. (2016). *Z. Kristallogr.* **231**, 209–217.

[bb17] Kaminsky, W. (2014). *WinTensor*. Version 1.5 for Windows. http://cad4.cpac.washington.edu/WinTensorhome/WinTensor.htm.

[bb18] Kim, J. S., Song, H. J., Roh, H. S., Yim, D. K., Noh, J. H. & Hong, K. S. (2012). *Mater. Lett.* **79**, 112–115.

[bb19] Liebau, F. (1985). *Structural Chemistry of Silicates*, p. 347. Berlin, Heidelberg, New York, Tokyo: Springer.

[bb20] Liu, H., Hildebrandt, E., Krammer, H., Kahlenberg, V., Krüger, H. & Schottenberger, H. (2021). *J. Am. Ceram. Soc.* **104**, 6678–6695.

[bb21] Liu, Q., Liu, Y., Ding, Y., Peng, Z., Yu, Q., Tian, X. & Dong, G. (2014). *J. Sol-Gel Sci. Technol.* **71**, 276–282.

[bb22] Mitchell, R. H. & Dawson, J. B. (2012). *Lithos*, **152**, 40–46.

[bb23] Momma, K. & Izumi, F. (2011). *J. Appl. Cryst.* **44**, 1272–1276.

[bb24] Morey, G. W. & Bowen, N. L. (1925). *J. Glass Technol. Soc.* **9**, 226–264.

[bb25] Napper, J. D., Layland, R. C., Smith, M. D. & Loye, H. (2004). *J. Chem. Crystallogr.* **34**, 347–351.

[bb26] Olanders, B. & Steenari, B. M. (1995). *Biomass Bioenergy*, **8**, 105–115.

[bb27] Orobengoa, D., Capillas, C., Aroyo, M. I. & Perez-Mato, J. M. (2009). *J. Appl. Cryst.* **42**, 820–833.

[bb28] Parauha, Y. R., Halwar, D. K. & Dhoble, S. J. (2022). *Displays*, **75**, 102304.

[bb29] Prince, E. (2004). Editor. *International Tables for X-ray Crystallography*, Vol. C, *Mathematical, Physical and Chemical Tables*, 3rd ed. Dordrecht: Springer.

[bb30] Reddy, P. M., Lakshmi, R., Dass, F. P. & Sasikumar, S. (2014). *Sci. Eng. Compos. Mater.* **23**, 375–380.

[bb31] Rigaku OD (2020). *CrysAlis PRO*. Rigaku Oxford Diffraction Ltd, Yarnton, Oxfordshire, England.

[bb32] Robinson, K., Gibbs, G. V. & Ribbe, P. H. (1971). *Science*, **172**, 567–570. 10.1126/science.172.3983.567 17802221

[bb33] Santoso, I., Riihimäki, M., Sibarani, D., Taskinen, P., Hupa, L., Paek, M. K. & Lindberg, D. (2022). *J. Eur. Ceram. Soc.* **42**, 2449–2463.

[bb34] Santoso, I., Taskinen, P., Jokilaakso, A., Paek, M.-K. & Lindberg, D. (2020). *Fuel*, **265**, 116894.

[bb35] Segnit, E. R. (1953). *Am. J. Sci.* **251**, 586–601.

[bb36] Shahid, K. A. & Glasser, F. P. (1971). *Phys. Chem. Glasses*, **12**, 50–57.

[bb37] Shannon, R. D. (1976). *Acta Cryst.* A**32**, 751–767.

[bb38] Shelby, J. E. (2009). *Glass Science and Technology*, 2nd ed., p. 291. Cambridge: The Royal Society of Chemistry.

[bb39] Sheldrick, G. M. (2008). *Acta Cryst.* A**64**, 112–122. 10.1107/S0108767307043930 18156677

[bb40] Skrzat, Z. M., Simonov, V. I. & Belov, N. V. (1969). *Dokl. Akad. Nauk SSSR*, **184**, 337–340.

[bb41] Varshneya, A. K. (1994). *Fundamentals of Inorganic Glasses*, p. 570. London: Academic Press.

[bb42] Weidendorfer, D., Schmidt, M. W. & Mattsson, H. B. (2016). *Contrib. Mineral. Petrol.* **171**, 43.

[bb43] West, A. R. (1978). *J. Am. Ceram. Soc.* **61**, 152–155.

[bb44] Williamson, J. & Glasser, F. P. (1965). *Science*, **148**, 1589–1591. 10.1126/science.148.3677.1589 17769914

[bb45] Wills, A. (2010). *VaList*. http://fermat.chem.ucl.ac.uk/spaces/willsgroup/software/.

[bb46] Wu, Q., Zhao, Q., Zheng, P., Chen, W., Xiang, D., He, Z., Huang, Q., Ding, J. & Zhou, J. (2020). *Ceram. Int.* **46**, 2845–2852.

[bb47] Zandi Karimi, A., Rezabeigi, E. & Drew, R. A. L. (2018). *J. Non-Cryst. Solids*, **502**, 176–183.

[bb48] Zhang, Z., Xiao, Y., Voncken, J., Yang, Y., Boom, R., Wang, N. & Zou, Z. (2011). *J. Am. Ceram. Soc.* **94**, 3088–3093.

