# Supplementary material for: Rb2Ca2Si2O7: a new alkali alkaline-earth silicate based on [Si2O7]6− anions
Source: Acta Crystallogr C Struct Chem. 2025 Feb 17;81(Pt 3):146–55. doi: 10.1107/S2053229625001196 (PMC11881166; doi:10.1107/S2053229625001196)
Supplement: Supplementary file 4 [file c-81-00146-sup4.pdf]

Acta Cryst C STRUCTURAL CHEMISTRY

Volume 81 (2025)

Supporting information for article:

$Rb_2Ca_2Si_2O_7$: a new alkali alkaline-earth silicate based on $[Si_2O_7]^{6-}$ anions

Volker Kahlenberg

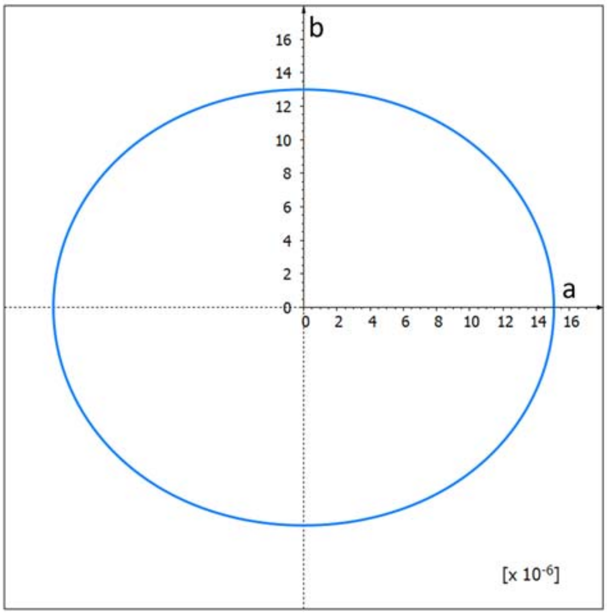
b
16
14
12
10
8
6
4
2
0
a
0 2 4 6 8 10 12 14 16
[x 10^{-6}]

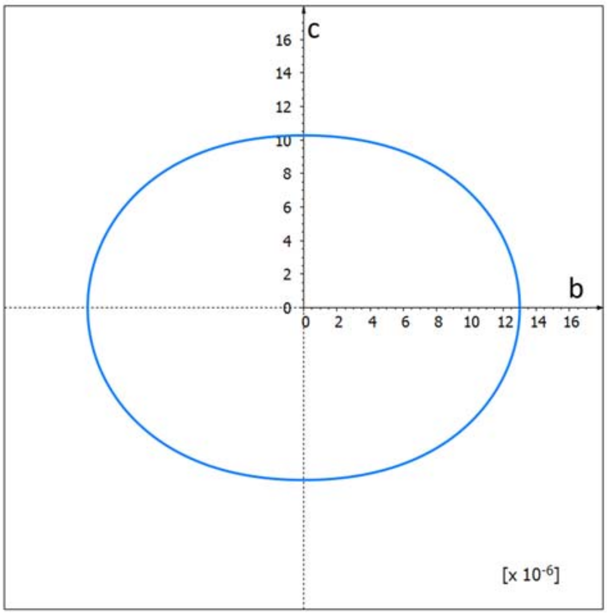
c
16
14
12
10
8
6
4
2
0
b
0 2 4 6 8 10 12 14 16
[x 10^{-6}]

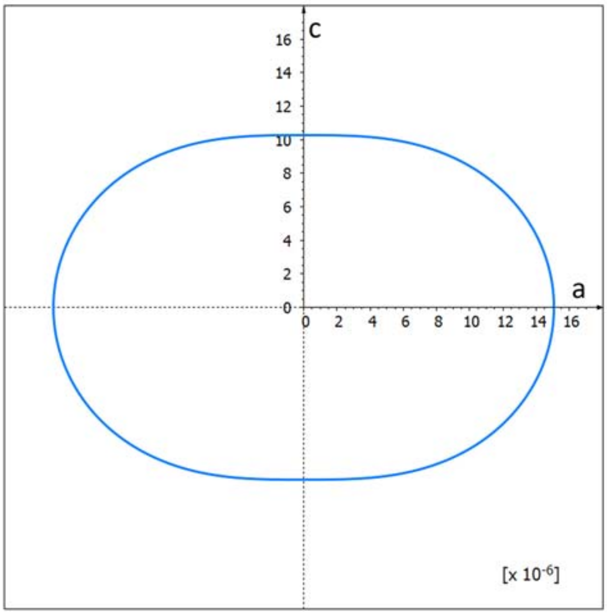

Figure S1 Sections through the three-dimensional representation surface of the thermal expansion tensor α_{ij} perpendicular to the principal axes: (*top*) **a**-**b**-plane, (*middle*) **b**-**c**-plane, and (*bottom*) **a**-**c**-plane.

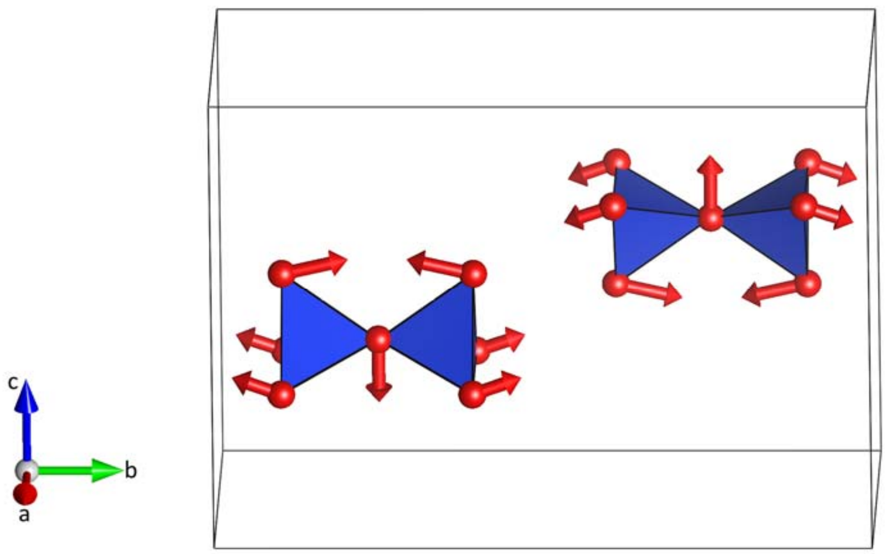

Figure S2 Displacement vectors belonging to the irrep M_4^- of the oxygen atoms of two [Si_2O_7]-groups of the parent phase (barycentres at *z* = ¼ and ¾, respectively). For the sake of clarity, the shift vectors have been scaled by a factor of two.
